# Fluctuations in Corneal Endothelial LAP2 Expression Levels Correlate with Passage Dependent Declines in Their Cell Proliferative Activity

**DOI:** 10.3390/ijms23105859

**Published:** 2022-05-23

**Authors:** Eleonora Maurizi, Alessia Merra, Davide Schiroli, Benedetta Ghezzi, Claudio Macaluso, Graziella Pellegrini

**Affiliations:** 1 Centre for Regenerative Medicine, University of Modena and Reggio Emilia, 41125 Modena, Italy; graziella.pellegrini@unimore.it; 2 Holostem Terapie Avanzate S.r.l., 41125 Modena, Italy; a.merra@holostem.com; 3 Transfusion Medicine Unit, Azienda USL-IRCCS, 42123 Reggio Emilia, Italy; davide.schiroli@ausl.re.it; 4 Dentistry Centre Lab, University of Parma, 43126 Parma, Italy; benedetta.ghezzi@unipr.it (B.G.); claudio.macaluso@unipr.it (C.M.)

**Keywords:** LAP2, corneal endothelium, fluctuating, marker, confluent, proliferation

## Abstract

The corneal endothelium is the inner corneal mono-layered epithelium, fundamental for preserving corneal hydration and transparency. However, molecular mechanisms that regulate corneal endothelial cells (CEnCs), in particular regarding their proliferative capacity, have been only partially elucidated. CEnCs are quiescent in vivo and they easily undergo endothelial to mesenchymal transition (EnMT) in vitro. This study aims to analyze CEnCs behavior and expression in vitro, either in sub-confluent growing (S) or confluent (C) CEnCs cultures. Primary rabbit and human CEnCs were cultured and used for RT-PCR, immunofluorescence or western blot analysis. These methods allowed identifying a novel molecular marker, LAP2, that is upregulated in S while downregulated in C human or rabbit CEnCs. Those results were observed for several subsequent passages in culture and this, together with the correlation between ki67 and LAP2 expression, suggested LAP2 as a novel possible indicator for culture ageing. Finally, treatment with FGF and TGFβ in rCEnCs highlighted how LAP2 can vary as the cells regulate their proliferative state. In conclusion, we have identified a novel marker for CEnCs, LAP2, that regulates its expression depending on the cells sub/confluent state and that correlates with CEnCs proliferation.

## 1. Introduction

The corneal endothelium (CE), the inner mono-layered epithelium of the cornea, retains the fundamental role of regulating corneal hydration and nourishment. Its dysfunction is responsible for corneal swelling and opacification, hence severely impairing correct vision. The state of the art for treating CE damages is corneal transplantation, the most frequent type of transplantation performed worldwide [1] and for which CE impairment represents the main indication. However, it is a complex surgical procedure, further limited by shortage of donor corneas and a considerable risk of graft detachment or allogenic rejection [1]. As a result of the long waiting list for corneal transplantation (only 1 in 70 people in need are covered, with 12.7 million people awaiting) [2], researches are seeking novel more effective treatments based on regenerative medicine.

Regenerative medicine approaches were previously demonstrated successful for other ophthalmological therapies, in particular for treatment of limbal stem cell deficiency (LSCD) that completed clinical trial and obtained marketing authorization from the European Medical Agency [3]. Reaching this goal requires a rigorous scientific evaluation of quality, safety and efficacy parameters that guarantee beneficial and standardised clinical outcomes. Quality assessment for in vitro cell culture includes a precise evaluation of cell morphology, tissue architecture and gene expression, analysing either specific markers for a particular tissue (cell identity) or markers associated with intracellular processes such as stemness, proliferation, epithelial to mesenchymal transition (EMT).

Corneal endothelial cells (CEnCs), differently than the limbal stem cells of the corneal epithelium, retain scarce proliferative potential: when subjected to significant loss due to iatrogenic damage or diseases in vivo, CEnCs enlarge and spread to cover the affected area [4]. This repairing mechanism fails when CEnCs density decreases below 500 cell/mm^2^ approximately, with CE becoming unable to maintain its pump function, degenerating into corneal oedema and eventually loss of vision. The CEnCs limited proliferative capacity is also preserved in vitro: several recent studies have worked at optimizing the culture protocol to maintain the correct human (H)CEnCs morphology and gene expression while expanding in culture [5,6,7]. The advancements in defining an optimal CEnCs culture protocol led to the first-in-human clinical trial by Kinoshita’s group [8], which employed HCEnCs from young donors, passaged two or three times in culture. HCEnCs at later passages (after the second-third) or from older donors easily lose their typical sealed hexagonal phenotype in vitro, undergoing endothelial to mesenchymal transition (EnMT) and senescence [9].

Beside a morphological characterization, a precise phenotypical and genotypical signature must define HCEnCs in order to develop a solid tissue engineering-based approach. Markers generally used to characterise CEnCs identity include ZO-1, Na^+^K^+^ATPase, Collagen8 [1], SLC4A11 [9] and, more recently, CD166 that is found specifically in HCEnCs [1]. In parallel, markers such as alpha-SMA [10] or CD44 [9] and CD73 [11] are deemed detrimental as associated with EnMT in CEnCs, and should be absent. Although identity markers used to date have been useful for an initial characterization, intracellular processes that regulate CEnCs physiology and proliferation in vitro across several passages have not yet been exhaustively described by associated markers [9]. To date, while 30% of the articles in CE are already proposing new therapies, only 11% are focused in finding new markers [12]. Considering that CE therapies based on regenerative medicine are not classified as surgical procedures but advanced therapy medicinal products, as cells are extensively manipulated in culture [12], it becomes crucial to expand and validate the panel of markers to assess the quality of cultured CEnCs. A tissue-engineered product entails in fact high regulatory standards of quality and safety if the product satisfies the criteria to enter the market as an effective therapy.

Understanding how CEnCs are regulated through variation of marker expression is important not only to guarantee regulatory standards related to cell therapies but also to develop alternative approaches, i.e., for inducing/inhibiting pathways that stimulate CE regeneration directly in vivo or during storage in eye banks, thus improving donor corneal quality and availability.

Herein we focused our attention on LAP2 (lamina-associated polypeptide 2), a marker associated with proliferation and senescence in different cell types [13], in particular in epithelial cells [14,15,16]. In CEnCs LAP2 was found to be upregulated in a sub-confluent (S) condition, whenever CEnCs are more actively proliferating, while in a confluent (C) state, when CEnCs have formed cell-cell contacts and reduce their proliferation, LAP2 is downregulated. This LAP2 up-downregulation kept repeating at subsequent passages but at lower extent as the passages increased, indicating a possible decrease in the cells capacity to maintain this physiologic mechanism. The results obtained show a positive correlation between LAP2 expression and proliferation which is altered over passages. According to this observation, we propose LAP2 as a novel marker of CE correct proliferation and cell culture status.

## 2. Materials and Methods

### 2.1. Ethical Statement

Human corneas, non-suitable for transplantation, were obtained from Italian Eye Banks with written informed consent from donor’s next of kin. Experimental protocol was approved by ISS-CNT (Italian National Transplant Centre): a national health authority managing the national procedures and rules regarding all Italian transplants and delegating the Tissue Banks to collect the written informed consents. The research protocol on human corneal tissues was approved by the local ethical committee (Comitato Etico dell’Area Vasta Emilia Nord, p. 0002956/20).

### 2.2. Corneal Endothelial Cell Culture

White New Zealand rabbits (3 months old, equivalent to 10 years of human age) were obtained from a local slaughterhouse (Maini SNC Modena) and rabbit corneas were harvested and processed within 24 h from euthanization. Descemet’s membrane was peeled off the cornea and rCEnCs isolated with Accutase (ECB3056D, Euroclone, Milan, Italy) for 20 min at 37 °C. rCEnCs were then pelleted at 1200 rpm for 3 min and plated in 6 well plates coated with FNC Coating Mix (AthenaES, Baltimore, USA). Growth medium was composed of OptiMEM-I (Thermo Fisher Scientific, Waltham, MA, USA), 8% HyClone fetal bovine serum (FBS; Fisher Scientific, Waltham, MA, USA), 5 ng/mL epidermal growth factor (EGF; Thermo Fisher Scientific, Waltham, MA, USA), 20 μg/mL ascorbic acid (Sigma-Aldrich, St.Louis, MI, USA), 200 mg/L calcium chloride (Sigma-Aldrich, St. Louis, MI, USA), 0.08% chondroitin sulphate (C4384, Sigma-Aldrich, St. Louis, MI, USA), and penicillin/streptomycin (Euroclone, Milan Italy). rCEnCs were cultured at 37 °C in 5% CO_2_, and the medium was changed every 2 days. Upon confluency, the rCEnCs were rinsed in DPBS and passaged at ratio of 1:2 or 1:3 with TrypLE (Thermo Fisher Scientific, Waltham, MA, USA) for 10–15 min at 37 °C in 5% CO_2_. Sub-confluent cultures were harvested 24 h after plating.

Human corneas tissues that fulfil quality criteria (age ranging from 49 to 78 years old, no history of corneas diseases, HCEnCs density greater than 1800 cells/mm^2^, death to preservation interval lower than 15 h) were preserved in Eusol at 4 °C and used within 15 days from death. HCEnCs were isolated following Descemet’s stripping and digestion with 1.5 mg/mL Collagenase A (Roche Diagnostic, Basel, Switzerland) for 3 h at 37 °C and then TrypLE for 5 min at 37 °C. HCEnCs were then pelleted at 1200 rpm for 3 min and plated in wells treated with FNC Coating mix.

### 2.3. Quantitative Real Time (RT)-PCR

RNA, extracted from CEnCs through the RNeasy Plus Micro Kit (Qiagen), was quantified with the Nanodrop 100 (Thermo Fisher Scientific, Waltham, MA, USA) and reverse transcribed into cDNA using the High Capacity cDNA Reverse Transcription Kit (Thermo Fisher Scientific, Waltham, MA, USA). RT-PCR assays were performed using 7900HT Fast Real-Time PCR System (Thermo Fisher Scientific, Waltham, MA, USA). SyBr Green technology (SyBr Green PCR MasterMix, 4309155, Thermo Fisher Scientific, Waltham, MA, USA) was used to distinguish the two LAP2 isoforms with the following primers (Table 1).

Data were normalised for ΔCt and ΔΔCt calculations using *GAPDH* as housekeeping control. For each condition, all complementary cDNA samples were run in triplicate. Three different strains were used for rabbit primary cultures at different passages (P0-P6, S-C) and three for tissues (T); three human tissues (T) and two strains of primary HCEnCs (P0-P3, S-C) were used for RT-PCR analysis.

### 2.4. Immunofluorescence (IF)

Immunofluorescence staining was performed after fixation in 3% PFA 15 min at room temperature (RT). Permeabilization was obtained with Triton X-100 (Bio-Rad, Hercules, CA, USA) 1% for 10 min at RT and blocking solution composed of bovine serum albumin (BSA; Sigma-Aldrich, St. Louis, MI, USA) 2%, FBS 2%, Triton X-100 0.01% in PBS for 30 min at 37 °C was used for saturation of non-specific binding sites. Primary and secondary antibodies were incubated for 1 h at 37 °C while nuclei were counterstained with DAPI (Roche Diagnostic, Basel, Switzerland) at RT for 5 min before mounting the glass coverslips using DAKO mounting medium (Agilent, Santa Clara, CA, USA). The primary antibodies used here were LAP2 1:100 (611000, BD biosciences, Franklin Lakes, NJ, USA), ki67 1:200 (ab15580, Abcam, Cambridge, UK), α-SMA 1:200 (A5228 clone1A1, Sigma-Aldrich, St. Louis, MI, USA). The secondary antibodies used are: Alexa Fluor 488 anti-rabbit, 1:2000, and Alexa Fluor 568 anti-mouse, 1:1000 (Thermo Fisher Scientific, Waltham, MA, USA). Images were obtained with a confocal microscope (LSM900 Airyscan—Carl Zeiss, Oberkochen, Germany). Six (C) and ten (S) fields from two different strains of two different biological experiments were used for quantification.

### 2.5. Growth Factors Treatment

rCEnCs (2.5 × 10^5^ cells) were seeded on an FNC-coated 6 well and treated at P1. FGF (Thermo Fisher Scientific, Waltham, MA, USA) and TGFβ (Miltenyi Biotec, Bergisch Gladbach, Germany) were used at a final concentration of 20 ng/mL and 10 ng/mL, respectively, in the culture medium. All the treatments described were performed 3 h after plating and the cells harvested 24 h after the treatment, as described previously [18].

### 2.6. Western Blot

The CEnCs analysed by western blot were collected following trypsinization, and proteins extracted using RIPA lysis buffer (R0278, SigmaAldrich, St. Louis, MI, USA), supplemented with protease and phosphatase inhibitors (97786, 78420, Thermo Fisher Scientific, Waltham, MA, USA). Proteins were then quantified through the Bradford assay (5000205—Bio-Rad, Hercules, CA, USA); equivalent amount of proteins were diluted in LDS Sample Buffer 4X and Sample reducing agent 10X (Thermo Fisher Scientific, Waltham, MA, USA), boiled for 10 min at 90 °C and loaded (100 V for 30 min and at 150 V for 1 h) in a 4–12% NuPAGE Bis-Tris Gels (Thermo Fisher Scientific, Waltham, MA, USA). Resolved proteins were then transferred to a nitrocellulose membrane (Bio-Rad, Hercules, CA, USA) for 2 h at 100 V at 4 °C, which was blocked afterwards for 1 h at room temperature using a blocking solution composed of 5% (*w*/*v*) non-fat milk in PBS supplemented with 0.05% Tween-20 (Bio-Rad, Hercules, CA, USA). The membranes were then probed with primary antibodies diluted in blocking solution: LAP2α (ab5162, Abcam, Cambridge, UK) 1:1000 and GAPDH (ab8245, Abcam, Cambridge, UK) at 1:10000, incubated overnight at 4 °C. Horseradish peroxidase-coupled secondary antibodies (sc-516102, Santa Cruz Biotechnology, Santa Cruz, CA, USA), used at 1:10,000 dilution for 1 h at room temperature, and a chemiluminescent substrate (SuperSignal West Pico, 34080, Thermo Fisher Scientific, Waltham, MA, USA) allowed visualization of the protein bands at the correct molecular weight through Chemi-Doc (Bio-Rad Hercules, CA, USA). The experiment was done in duplicate and HeLa cells were used as positive control for LAP2α band visualization (data not shown).

## 3. Results

### 3.1. LAP2 RNA Expression in Primary rCEnCs

**Figure 1:**
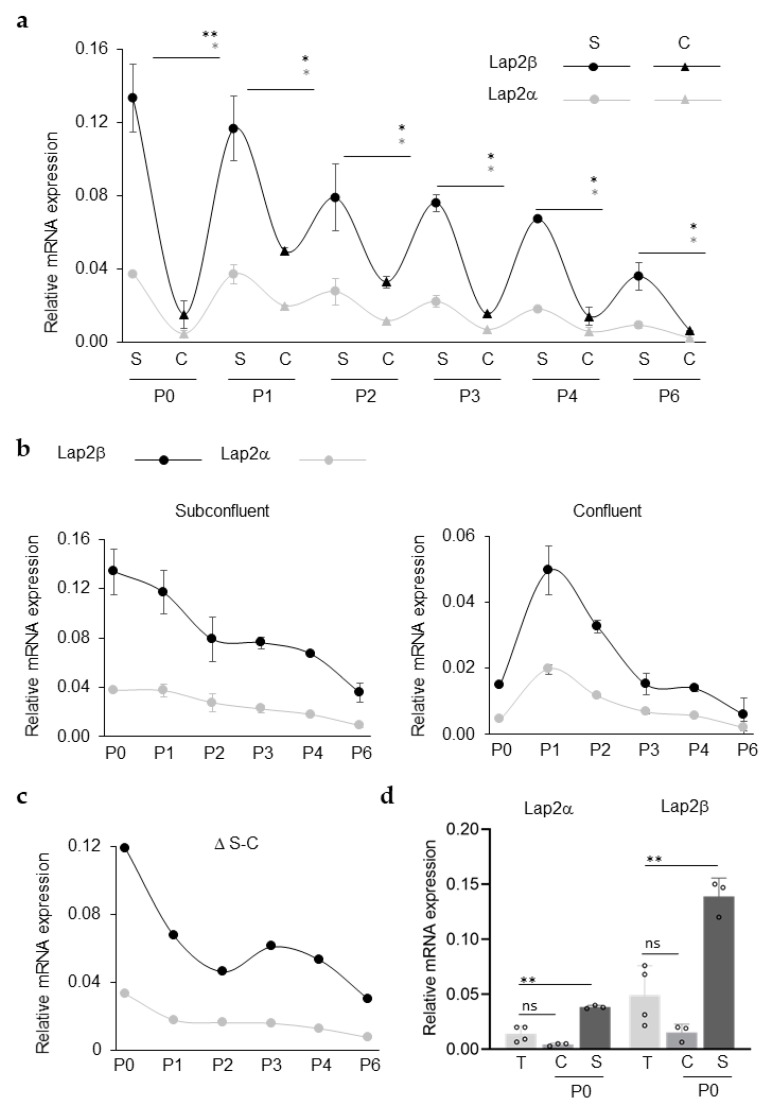
LAP2 is upregulated in sub-confluent rCEnCs (**a**). RT-PCR (Sybr Green) showing up and down-regulation of *LAP2α* and *β* across the passages in rCEnCs sub-confluent (S) and confluent (C) cultures. The *LAP2* ability of being up and down-regulated decreases with passages (**b**). RT-PCR, divided between S and C populations highlighted how *LAP2* expression decreases with passages till P6 when it is drastically reduced (**c**). Delta of *LAP2* expression between S and C populations, studied across passages (P1 to P6) (**d**). RT-PCR of *LAP2α* and *β* expression in rCEnCs isolated from the Descemet tissues (T), compared with rCEnCs in culture at P0. Data are presented as mean ± SE. The statistical significance was determined by Students’ *t*-test. * *p*-value < 0.05. ** *p* < 0.01.

Primary rCEnC were cultured in vitro from passage (P) 0 to P6 and RNA expression was evaluated for each subsequent passage, either when rCEnCs were sub-confluent (50% confluency) or confluent (100% confluency). Quantitative RT-PCR was used to determine the relative expression of the two most commonly studied isoforms of *LAP2*, alpha and beta [19]. In each passage, rCEnCs exhibited an increased expression of both *LAP2α* and *LAP2β* in the sub-confluent (S) condition, when compared with the confluent (C) cultures (Figure 1a). Moreover, in each sample, the beta-isoform was more expressed than the alpha isoform. At P6, both *LAP2α* and *LAP2β* decrease their expression to a value close to zero. Analysing *LAP2* expression in S and C cultures separately (Figure 1b), it was clear that the *LAP2α* and *β* expression levels decreased gradually from the initial to the late passages in the S group of samples, drastically dropping at P6 (Figure 1b, left). In the C group (Figure 1b, right), we observed a gradual decrease in *LAP2α* and β expression starting from P1. Furthermore, considering the difference between S and C cultures (Figure 1c), the values of the two isoforms are decreasing rapidly at the first passages and then more gradually at each following passage from P2-P3. *LAP2* expression was also evaluated in rCEnCs isolated from the tissue (T), showing a low expression for both isoforms if compared with the expression registered at P0 S (Figure 1d).

### 3.2. LAP2 and ki67 Protein Expression in Primary rCEnCs

**Figure 2:**
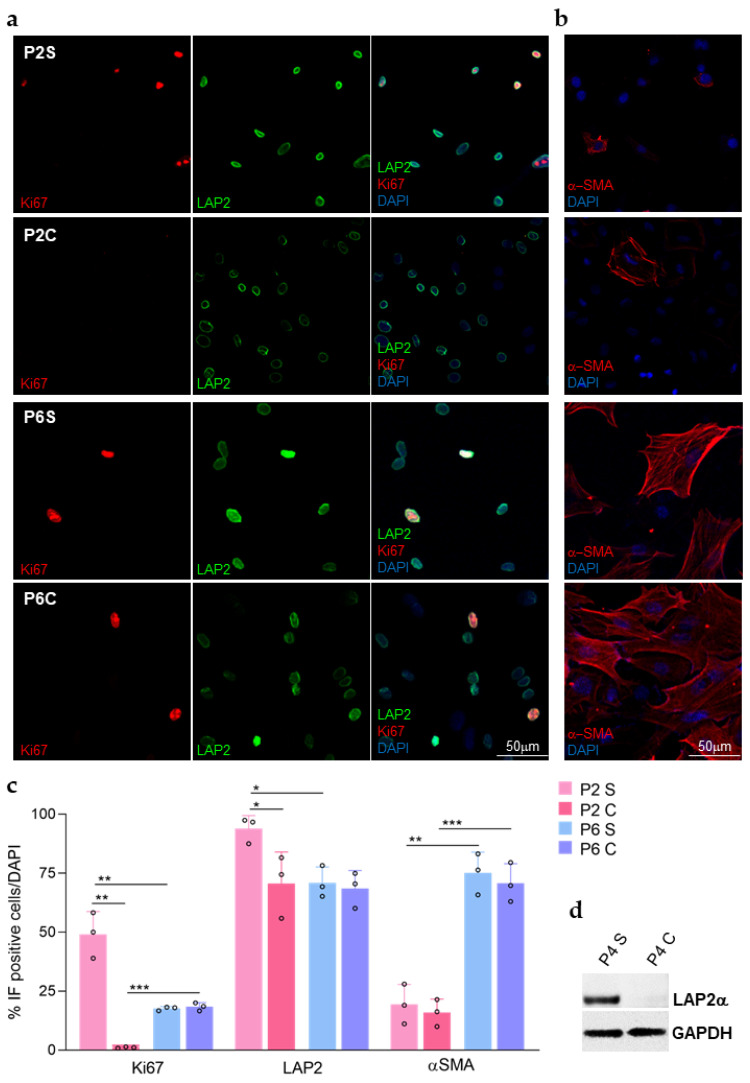
LAP2 protein increases in rCEnCs at S only at low passages. (**a**) Representative images of ki67 (red) and LAP2 (green) immunofluorescence staining of rCEnC at P2 S_C and P6 S_C. DAPI (blue) counterstained nuclei. (**b**) Representative images of αSMA (red) immunofluorescence staining of rCEnC at P2 S_C and P6 S_C. DAPI (blue) counterstained nuclei. (**c**) Quantification of immunofluorescence staining shown in (**a**,**b**). Data are presented as mean ± SE. The statistical significance was determined by Students’ *t*-test. * *p*-value < 0.05. ** *p* < 0.01. *** *p* < 0.001. (**d**) Western Blot analysis showing LAP2α expression on rCEnC P4 S_C. GAPDH was used as internal control.

LAP2 expression was further evaluated at protein level by immunocytochemistry, together with expression of the ki67 proliferation marker (Figure 2a). Quantification of immunofluorescence analysis on rCEnCs allowed observing that at low passages (P2) LAP2 protein expression decreased significantly at C (*p* < 0.001), if compared with the same cells at the same passage but at S (Figure 2a,c). This LAP2 protein modulation between S and C was confirmed by western blot (Figure 2d) and reflects RT-PCR measured changes in mRNA expression levels (Figure 1). In parallel, ki67 expression confirmed that the cells were actively proliferating at S, when they showed a significantly higher LAP2 expression if compared with the C counterpart (Figure 2a,c). If we consider the ratio between ki67 and LAP2 at P2 (ratio ki67/LAP2: 46.3 ± 7.7 at S and 1.65 ± 0.6 at C) and P6 (ratio ki67/LAP2: 26 ± 3 at S and 23.6 ± 3.8 at C), the difference between S and C disappears at high passages (P6). Similarly, the delta both in ki67 and LAP2 expression in S and C cultures disappeared at P6 (Figure 2a,c). The lack of difference in LAP2 expression between S and C cultures at P6 again reflects what was previously observed in mRNA expression. Moreover, the same level in S and C groups of ki67 at P6 underlies how the proliferative control is altered at high passages in both conditions. The high levels of αSMA protein expression, a marker of EnMT, which is similar at P6 in S and C group but much lower at P2, either at S or C (Figure 2b) suggests that this alteration might be due to an impaired cell-cell contact inhibition.

### 3.3. TGFβ-FGF Treatment in Primary rCEnCs

**Figure 3:**
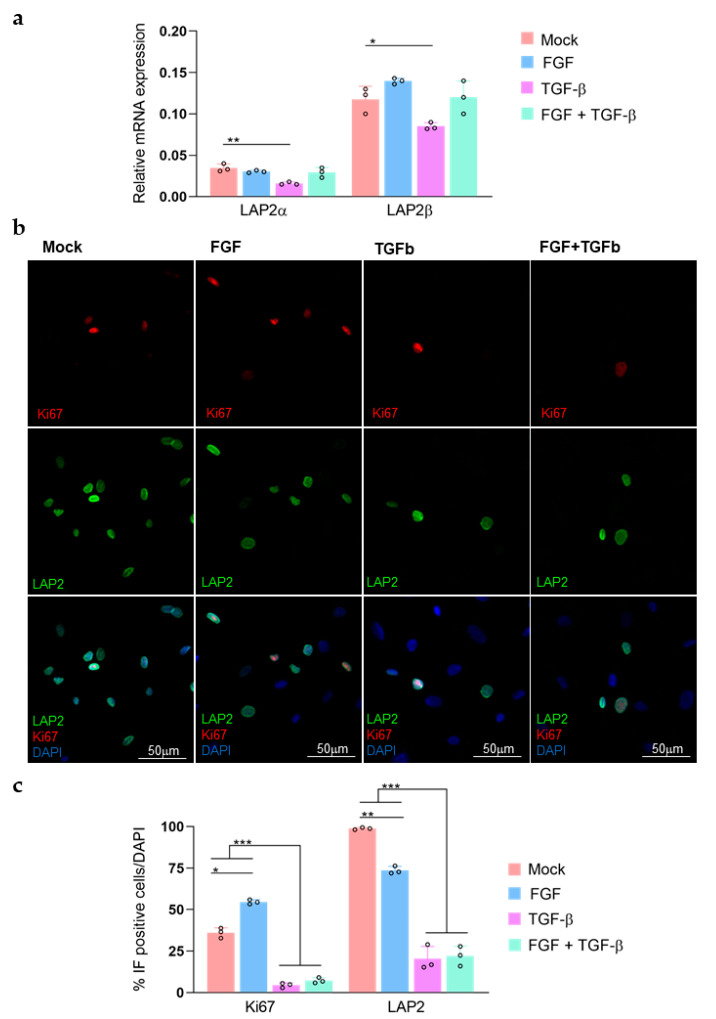
LAP2 regulation upon FGF and TGFβ treatments (**a**) RT-PCR (Sybr Green) showing up and down-regulation of LAP2α and LAP2β in rCEnCs (P1) following treatments with FGF and TGFβ. (**b**) Representative images of ki67 (red) and LAP2 (green) immunofluorescence staining of rCEnCs at P1 upon FGF and TGFβ treatments. DAPI (blue) counterstained nuclei. (**c**) Quantification of immunofluorescence staining shown in (**b**). Quantification data are presented as mean ± SE. The statistical significance was determined by Students’ t-test. * *p*-value < 0.05. ** *p* < 0.01. *** *p* < 0.001.

To better understand how variations in LAP2 expression levels are related to modulation of cell proliferative status, we treated rCEnCs with TGFβ and FGF (Figure 3). When compared to the Mock control, the TGFβ treated cells underwent highly significant decline in RT-PCR measured *LAP2α* and *LAP2β* RNA expression levels (Figure 3a). Similarly, TGFβ treatment induced significant reductions in both ki67 (*p* < 0.001) and LAP2 (*p* < 0.001) in rCEnCs (Figure 3b,c). A similar decrease either in ki67 or LAP2 protein expression was observed in rCEnCs treated with TGFβ and FGF simultaneously (Figure 3b,c). Whenever the cells were treated with FGF only, we detected an increase in ki67 expression, as expected [18,20]. A parallel decrease in LAP2 protein expression levels was observed in FGF treated rCEnCs (Figure 3b,c).

### 3.4. Primary Human (H)CEnCs and LAP2

**Figure 4:**
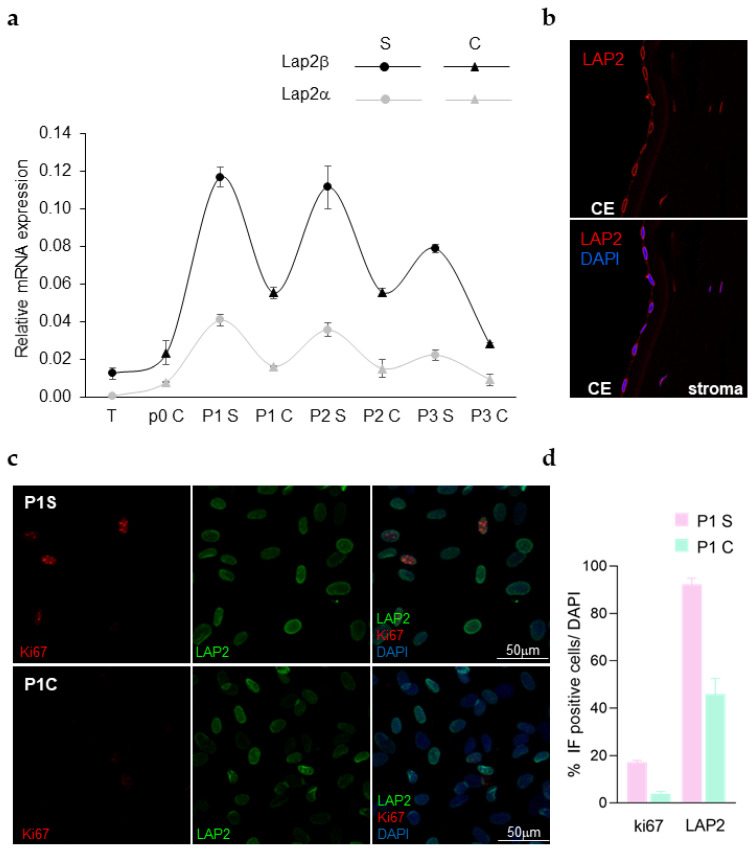
LAP2 expression in human CE (**a**) RT-PCR (Sybr Green) showing up and down-regulation of *LAP2α* and *β* in HCEnCs isolated from the tissues (T) and at subsequent passages (P0-P3) in S and C HCEnCs culture in vitro. (**b**) Representative images of LAP2 (red) immunofluorescence staining in cryosections of the human cornea, showing CE and stroma. DAPI (blue) counterstained nuclei. (**c**) Representative images of LAP2 (green) and ki67 (red) immunofluorescence staining on HCEnCs in vitro, at P1. DAPI (blue) counterstained nuclei. (**d**) Quantification of the immunofluorescence data shown in (**c**).

LAP2 expression was further investigated in HCEnCs (Figure 4). The RNA expression was evaluated both in the HCEnCs isolated from the tissue and in a primary culture at subsequent passages (Figure 4a). A pattern resembling the one identified in rCEnCs (Figure 1a) was observed in S (elevated *LAP2* expression) and C (low levels of *LAP2*) HCEnCs cultures, either for the *LAP2α* or *LAP2β* isoforms. A basal level of *LAP2* RNA expression was observed in cells directly isolated from human tissues (T), expression that was then detected also at protein level in HCEnCs nuclear membrane in corneal cryosections (Figure 4b). Conversely, in primary HCEnCs in vitro, the LAP2 signal was found diffused across the whole nuclei, higher in S than C cultures (Figure 4c,d). In parallel, similarly to what was previously observed in rCEnCs, ki67 showed a higher expression in S than C HCEnCs (Figure 4c,d).

## 4. Discussion

Nearly 13 million people worldwide are awaiting a corneal transplantation, for which CE impairment represents the main indication [2]. For decades researchers have been seeking the key to induce CEnCs to proliferate, which is the principal hindrance that still hampers the clinical application of a regenerative strategy to repair CE damages [1]. However, CEnCs proliferation has not been described to date by expression of specific markers: CEnCs characterization markers commonly used are not tissue specific and not clearly associated to CE intracellular processes [12]. Description of novel markers involved in CE functional pathways such as phenotypical changes and proliferative capacity becomes therefore a primary concern towards the development of a functional innovative regenerative product that fulfils the regulatory quality and safety criteria.

A protein involved in proliferation in multiple tissues, including epithelial tissue, is LAP2 [21]: a family of six isoforms resulting from alternative splicing of the same gene [22], which is highly conserved among mammals [23]. LAP2 proteins mediate attachment of chromatin to the nuclear membrane and LAP2α and β are the best-characterised isoforms: they share the N-terminal region but, while LAP2β is anchored to the nuclear membrane, the LAP2α isoform is diffused throughout the nucleoplasm [23].

Proliferating cells of tissues characterised by an elevated regenerative capacity such as skin, thymus, testis and ovary exibited a high LAP2β expression [24]. Similarly to LAP2β [24], LAP2α was found expressed in early progenitor cells of proliferating tissues like epidermis, colon, skeletal muscle and preadypocytes [13,21,25]. These results were confirmed by Parise and co-workers, correlating an elevated LAP2α with proliferation in tumours [26]. All those pieces of evidence underlie an essential role for LAP2 expression levels during the proliferation process.

Herein we describe LAP2 as a novel marker expressed in human and rabbit CE, either in vivo or in vitro. In CEnCs we have observed a fluctuating expression of both LAP2α and LAP2β in rCEnCs, depending on the confluency state in culture (Figure 1) and proliferation (Figure 2). Whenever the cells were actively proliferating (S, high ki67; Figure 2a,c) the LAP2 expression level was significantly higher than when proliferation stopped (C, low ki67 expression at P2; Figure 2a,c). Intriguingly, all the ki67 expressing CEnCs were also LAP2 positive (Figure 2a and Figure 3b). ki67 protein levels rise throughout cell cycle phases, reaching the highest peak during mitosis [27], while LAP2 seems to be expressed during mitosis together with ki67 but also post-mitotically in the ki67-negative cells, as suggested by Vlcek et al. [28].

Differently from the actively proliferating CEnCs, the cells isolated from the tissue, which are in a quiescent stage in vivo [1], demonstrated very low levels of LAP2α and β. (Figure 1d). Similarly, Pekovic et al. described a LAP2α upregulation whenever cells are entering G_1_ phase and a downregulation when the cells are quiescent in G_0_ [29]_._ This was further confirmed by a decreased LAP2 expression, both at RNA and protein level (Figure 3), observed in CEnCs treated with TGFβ, which was proved to reduce proliferation (Figure 3b, low ki67) [18,30,31].

As a monolayer, CEnCs in culture alternate a proliferative phase and a mitotic arrest at confluence following contact inhibition [32]. The CEnCs mitotic block occurs at G_1_ phase of the cell cycle [33], while they re-enter the cell cycle through G_2_/M when at sub-confluence: rCEnCs, whenever at 60% confluence, presented a 20% of cells actively proliferating in G_2_/M of cell cycle [18]. The succession of those proliferative and not-proliferative phases reflects the fluctuating trend observed in LAP2 expression, either in CEnCs or in other cell types [21]. In particular, at P0 S the LAP2 expression increased to the highest level registered if compared with the S stage at the following passages (Figure 1a,b), suggesting the need for an initial “boost” of transcript in order to allow the cell cycle progression. The rapid decrease in Δ S-C seen at P1 (Figure 1c) may indicate that *LAP2* up-downregulation in CEnCs attenuates already at the first passages in culture. The Δ S-C decrease at P1 is due to an increase of LAP2 expression either at C (Figure 1b, right) or at S if compared with P0: we can speculate that the cells start losing the dynamic LAP2 regulation promoted by the succession of proliferative and non-proliferative phases, flattening the curve to become close to zero. The lack of the same regulatory system observed at P0 is exacerbated at high passages, when the sub confluent (S) to confluent (C) statusprogressively decrease (Figure 1c).

At high rCEnCs passages, differently from the early ones, ki67 and LAP2 expression was low in proliferating cells (Figure 2, P6S). This is possibly due to the increase of a senescence process, during which LAP2 expression decline naturally as in myoblasts [13] but also in primary human dermal fibroblasts and human skin keratinocytes in vitro and in vivo [14,34]. Moreover, while ageing (P1 > P6), CEnCs encountered a variation of Δ S-C in *LAP2* expression which lowered progressively (Figure 1c). Concomitantly, a decreased variation between S and C at high passages is seen also for ki67 expression (Figure 2): the proliferation decreased at S while it increased at C, if compared with the early passages. This suggests the presence of a double process occurring at high passages: ageing (decreased proliferation at S) and EnMT (increased proliferation at C). The progression towards an EnMT phenotype at high passages in CEnCs, as documented either for human or rabbit CEnCs in the literature [9,18,35], is highlighted by an increased αSMA expression (Figure 2b).

In accordance with these observations in CEnCs, intracellular processes such as quiescence, senescence or differentiation have already been associated to a decreased LAP2 expression [13,21]. This further reveals a role of LAP2 in balancing proliferation and cell cycle exit. A dynamic regulatory complex was proposed that is responsible for cell genomic plasticity during proliferation while, upon differentiation, quiescence or senescence, this regulation might be no longer required and thus lost by the cells, with a concomitant reduction in LAP2 expression levels [21].

To confirm this model, CEnCs proliferation was induced by adding FGF to the culture media (Figure 3). Whenever CEnCs are treated with FGF and proliferation increases (high ki67, Figure 3b) as previously known [18,20], we detected a parallel decrease in LAP2 expression only by immunofluorescence (Figure 3b,c), while no differences with Mock were observed at the RNA level (Figure 3a). The lacking increase in LAP2 despite the cells are proliferating can indicate that a FGF forced growth induction in rCEnCs might not represent the naturally occurring proliferative process we detected in S cultures. In fact, FGF generally promotes EnMT progression in CEnCs [18,35,36]: the decrease in LAP2 expression upon FGF could therefore resemble the LAP2 reduction observed at high passages (P6, Figure 1 and Figure 2), when EnMT is increasing (high αSMA expression, Figure 2b).

In HCEnCs we confirmed the *LAP2* expression results previously obtained in rCEnCs: LAP2 was upregulated in S and downregulated in C cultures, modulation that seems to decrease as passages increase (Figure 4a). In the human tissue in vivo, LAP2 delineated the HCEnCs nuclei, showing how the isoforms detected by our antibody appear to be confined mainly to the nuclear membrane whenever the cells are quiescent (Figure 4b). LAP2 protein evaluation confirmed a LAP2 downregulation in C cultures when compared with the S counterpart (Figure 4c,d). The parallel ki67 detection nearly disappeared in C cultures, indicating a mitotic arrest following contact inhibition (Figure 4c,d), which at P1 when the cells were analysed still associates with a lower LAP2 expression. However, differently from the rabbit samples, the human donors are highly heterogeneous: therefore, the data obtained with human CE, although highly promising, could be considered preliminary and need further confirmation in a larger cohort of HCEnCs, cultured in multiple conditions.

Considering the analysis of our cultures, we could describe here how the expression of LAP2, together with the more common ki67 or αSMA markers, vary across the passages. To date, no markers have been described for assessing the quality of an early CEnCs culture and LAP2 could be a good candidate that associates with CEnCs proliferation and ageing. Identifying key parameters, defining times and thresholds, and establishing how these can change during CEnCs culture, while correlating with tissue architecture and identity markers, is fundamental in the future perspective of developing a reproducible regenerative strategy for CE.

## 5. Conclusions

A new marker for CE, LAP2, which varies its expression depending on the CEnCs proliferative state has been here identified. LAP2 fluctuating expression during different cell culture phases can reveal fundamental insights to properly stimulate CEnCs regeneration with the final aim to delineate an effective CE therapeutic method.

## Figures and Tables

**Table 1:** Forward and Reverse primers used in SyBr Green RT-PCR for either rabbit or human samples. Amplicon size is indicated on the right column for each gene.

Gene	Specifications	Sequence (5′ to 3′)	Size (bp)
*LAP2α* and *β*	Forward_rabbit and Human	ATT GTG GGA ACA ACC AGG AA	
*LAP2α*	Reverse_rabbit	CCC TAG TGG ACT TCA CTT TCT	205
	Reverse_Human	CCA CCA GAG GGA GTA GTT C	248
*LAP2β*	Reverse_rabbit	CCC TTT AGC GGT TCT CTC T	212
	Reverse_Human	TTT GCT CTG CCC TTT AGT GG	221
*GAPDH*	Forward_rabbit	TGA CGA CAT CAA GAA GGT GGT G	120 [17]
	Reverse_rabbit	GAA GGT GGA GGA GTG GGT GTC	
	Forward_ Human	GTC TCC TCT GAC TTC AAC AGC G	131
	Reverse_ Human	ACC ACC CTG TTG CTG TAG CCA A	

## Data Availability

The data presented in this study are available on request from the corresponding author. The data are not publicly available due to privacy and ethical restrictions.
